# Dietary Nonstarch Polysaccharide Intake and Risk of Colorectal Cancer: Findings from the Singapore Chinese Health Study

**DOI:** 10.1158/2767-9764.CRC-22-0153

**Published:** 2022-10-31

**Authors:** Yi-Chuan Yu, Pedram Paragomi, Aizhen Jin, Renwei Wang, Robert E. Schoen, Woon-Puay Koh, Jian-Min Yuan, Hung N. Luu

**Affiliations:** 1 University of Pittsburgh Medical Center (UPMC) Hillman Cancer Center, Pittsburgh, Pennsylvania.; 2 Healthy Longevity Translational Research Programme, Yong Loo Lin School of Medicine, National University of Singapore, Singapore.; 3 Division of Gastroenterology, Hepatology, and Nutrition, Department of Medicine, University of Pittsburgh Medical Center, Pittsburgh, Pennsylvania.; 4 Department of Epidemiology, School of Public Health, University of Pittsburgh, Pittsburgh, Pennsylvania.; 5 Singapore Institute for Clinical Sciences, Agency for Science Technology and Research (A*STAR), Singapore.

## Abstract

Dietary fiber or nonstarch polysaccharides (NSP) may provide protection from colorectal cancer development. Epidemiologic studies on the association between dietary fiber and colorectal cancer is inconsistent are limited on NSP as a modifiable risk factor. Using the Singapore Chinese Health Study, a population-based prospective cohort of 61,321 cancer-free middle-aged or older Chinese Singaporeans, we examined the association between dietary fiber and NSP intakes and colorectal cancer risk. Fiber and NSP intakes at baseline were obtained using a validated semiquantitative food frequency questionnaire coupled with the Singapore Food Composition Database. Cox proportional hazard regression model was used to estimate the HRs and respective 95% confidence intervals (CI) for colorectal cancer associated with dietary fiber and NSP intakes after adjusting for potential confounders. After an average of 17.5 years of follow-up, 2,140 participants developed colorectal cancer. NSP was inversely associated with the risk of colorectal cancer in a dose-dependent manner, whereas dietary fiber was not associated with risk of colorectal cancer overall or histologic subtypes. The multivariable-adjusted HRs (95% CIs) of colorectal cancer for quartiles 2, 3, and 4 of dietary NSP intake were 0.99 (0.88–1.11), 0.98 (0.87–1.11), and 0.84 (0.73–0.95), respectively, compared with the lowest quartile (*P*_trend_ = 0.006). This inverse association was more apparent for colon cancer (HR_Q4 vs. Q1_ = 0.79, 95% CI: 0.67–0.93, *P*_trend_ = 0.003) than rectal cancer (HR _Q4 vs. Q1_ = 0.92, 95% CI: 0.74–1.13, *P*_trend_ = 0.53). Our findings suggested that dietary NSP but not fiber is associated with a reduced risk of colon cancer in Chinese Singaporeans.

**Significance:**

NSPs may be beneficial for colorectal cancer primary prevention.

## Introduction

Colorectal cancer contributes around 10% of annual global cancer–related deaths (1). More than 1.2 million people are diagnosed with colorectal cancer each year with an increasing trend (2). By 2035, new cases of colorectal cancer are projected to reach 2.5 million worldwide (1, 3). According to the 2018 data from the GLOBOCAN, colorectal cancer is the third leading cause of cancer mortality. The estimated age-standard incidence rate among Asians was 17.7 per 100,000 persons in 2018 (4) whereas the corresponding rates in Singapore were 39.3 per 100,000 persons in men and 27.3 per 100,000 persons in women (5), which were lower than those in the United States (i.e., 46.9 per 100,000 persons in men and 35.6 per 100,000 persons in women; ref. 6). Alcohol usage, red meat and/or processed/preserved meat intake, obesity, physical inactivity, and type 2 diabetes are established risk factors for colorectal cancer (7). Agents such as aspirin and calcium supplements have been shown to have protective effects against colorectal cancer development (8–10).

Dietary fiber was introduced by Burkitt (11) as a potential protective agent for colorectal cancer and derived from the low incidence rate of colorectal cancer in certain areas of Africa where the fiber consumption is relatively high. The definition of dietary fiber can be very broad (12). Briefly, dietary fiber is defined as part of the plants that are edible but not digestible in the human small intestine (13). The amount of dietary fiber intake varies in different diets: relatively lower in the western diet and higher in rural African diet (rural African communities may consume up to 7 times more fibers than western diet; ref. 12). Nonstarch polysaccharide (NSP) is a major subtype of dietary fiber and has a laxative effect (14). There are two types of NSPs: soluble NSP and insoluble NSP. Both soluble and insoluble NSP are nondigestible where soluble NSP increases the intestinal viscosity and insoluble NSP creates the bulk in the diet (15). As of 2022, several meta-analysis or prospective cohort studies (16–25), including the Nurses’ Health Study (NHS; ref. 17), the Health Professional's Study (HPFS; ref. 18) and the Multiethnic Cohort Study (MEC ref. 23), all in the United States, as well as the European Prospective Investigation into Cancer and Nutrition (EPIC; ref. 26), examined and reported inconsistent associations between dietary intake of fiber and/or NSP and risk of colorectal cancer. In addition, some of these studies found null associations between dietary fiber intake and the risk of developing colorectal cancer (17, 18, 21) with a few reported some protective effects in restricted demographic subgroups (23, 26) or particular foods groups containing fiber (16, 19, 20). On the other hand, a few studies reported negative associations between dietary NSP intake and the risk of colorectal cancer (24, 25). Clinical trials also failed to demonstrate a protective effect of dietary fiber against the development of colorectal cancer (27) or recurrence of colorectal adenomas (28). The observed inverse fiber–colorectal cancer risk association could be attributable to NSP which is derived from similar food sources and highly correlated with fiber. Because there are inconsistent findings in epidemiologic literature regarding fiber and colorectal cancer risk, limited study on NSP as a modifiable risk factor and limited research in Asian populations, additional studies in this particular population with different dietary habits may help discern a differential effect of fiber components on risk of colorectal cancer.

In the current analysis, we examined the association for intake of dietary fiber and NSP with the risk of colorectal cancer in the Singapore Chinese Health Study (SCHS), an ongoing prospective cohort study of more than 63,000 Chinese men and women in Singapore. The study population has unique dietary habits from western populations studied in previous studies.

## Materials and Methods

### Study Population

Data used for the current analysis was derived from SCHS. The study design, subject recruitment, and data collection were described in detail previously (29). Briefly, the SCHS is an ongoing prospective cohort study for cancer and other chronic diseases. A total of 63,257 Chinese men and women were enrolled between April 1993 and December 1998 when the subjects were 45–74 years old. The study participants were residents of the Singapore government-built housing and belonged to either Hokkien or Cantonese dialect groups, which were originated from Fujian and Guangdong provinces, respectively in Southern China. All study participants provided informed consents. All study participants provided written informed consent. The study was conducted in accordance with recognized ethical guidelines (e.g., Declaration of Helsinki, CIOMS, Belmont Report, U.S. Common Rule). The Institutional Review Boards (IRB) of the National University of Singapore and the University of Pittsburgh have continuously approved the SCHS.

### Dietary Assessment

Dietary assessment for each participant was completed using a validated semiquantitative food frequency questionnaire (FFQ) including 165 food items or groups that were commonly consumed by Chinese Singaporeans. Each participant was asked to choose from 8 frequency levels of food consumption from the “*never or hardly ever*” to “*two or more times a day*”. The information on the portion size of each food item was also collected with the assistance of a photo album showing the small, the medium, and the large portion sizes of a given food. The FFQ was validated against a series of 24-hour dietary recall surveys (30) as well as biomarker studies (31, 32). The average daily intake of 100 nutrients and non-nutrient compounds for each participant was calculated using the Singapore Food Composition Database and the correlation coefficients among most of the calorie-adjusted nutrients derived from the 24-hour dietary recalls and the FFQ ranged from 0.24 to 0.79 (30).

### Assessment of Other Covariates

Besides diet, the baseline questionnaire also asked participants for information on demographics, body weight and height, alcohol consumption, tobacco smoking, current physical activity, occupational exposure, medical history, family history of cancer, and reproductive history (women only). Weekly hours of physical activity were grouped in 8 different levels: (i) never, (ii) 0.5–1, (iii) 2–3, (iv) 4–6, (v) 7–10, (vi) 11–20, (vii) 21–30, and (viii) 31 hours or more. Three common physical activities were included: (i) strenuous sports (i.e., jogging, cycling on hills, tennis, swimming, tennis, or aerobics); (ii) vigorous work (i.e., moving heavy items, shoveling, or related labor work); and (iii) moderate activities (walking, cycling on flat ground, Tai Chi, and Chi Kung; ref. 33). Alcohol drinking was grouped into non-heavy drinker or heavy drinker, which was defined as 15 drinks/week or more for men and 8 drinks/week or more for women according to the most recent guideline by the US Center for Disease Control and Prevention (CDC; ref. 34). BMI was calculated as weight divided by height in meters and overweight (23–<27.5 kg/m^2^) or obesity (≥27.5 kg/m^2^) were defined following the recommendation from the World Health Organization (WHO) for Asian populations (35, 36).

### Assessment of Dietary Fiber and NSP

The dietary fiber and dietary NSP in this study were derived from their respective contents in commonly consumed food items reported in the FFQ using residual method adjusting for energy intake (37). Overall there were 17 food items commonly consumed by Chinese Singaporeans: (i) red meat; (ii) poultry; (iii) fish and shellfish; (iv) eggs; (v) total legumes; (vi) vegetable, fruits, or related juice; (vii) grain products; (viii) dairy products; (ix) desserts; (x) nuts and seeds; (xi) bread spread; (xii) fats and cooking oil; (xiii) sugar and candy; (xiv) non-dairy beverages; (xv) sauces and condiments; (xvi) flour and baking ingredients; and (xvii) soup stock and canned soup. Major contributors to dietary fiber were grain products (31%), fruits and their related juice (28%), and vegetables and their related juice (21%). Grain products, fruits and their related juice, and vegetables and their related juice products accounted for 24%, 28%, and 24%, respectively, of total insoluble NSP. The corresponding proportion of soluble NSP by these food items were 21%, 41%, and 21% (Supplementary Table S1).

### Ascertainment of Colorectal Cancer Cases

The incident cases of cancer and death among the study participants were identified through annual record linkage analyses of all surviving cohort participants with the nationwide Singapore Cancer Registry and the Singapore Registry of Birth and Deaths, respectively. The IRB cases were defined by the International Classification of Disease-Oncology Second Edition (ICD-O-2) C18-C20 (C18 for colon cancer and C19-C20 for rectal cancer; ref. 38). The follow-up for cancer incidence and vital status of the cohort participants has been virtually complete as to date, overall only 56 original participants (<0.1%) were lost to follow-up due to their emigration out of Singapore.

### Statistical Analysis

In this analysis, 1,936 participants with a history of cancer at baseline were excluded, leaving 61,321 subjects included in the final analysis. We used the *t* test for continuous variables and the *χ*^2^ test for categorical variables to compare baseline characteristics among different categories of dietary fiber and NSP intake and between cases and noncases.

Person-years for each study participant at risk was counted from the time of baseline interview to the time of colorectal cancer diagnosis, emigration out of Singapore, death, or December 31, 2015, whichever occurred first. Study subjects were grouped into quartile levels of dietary fibers or NSP intake. HRs and their 95% confidence intervals (CI) of colorectal cancer were calculated for quartiles 2–4 of dietary fibers or NSP intake compared with the lowest quartile. A linear trend tests were performed on the median values of each quartile due to the skewness of fiber and NSP intake. The proportional hazards assumption in the Cox regression model was examined and confirmed without violation using Schoenfeld residuals. Potential confounders in the Cox proportional hazard regression models were age (years), sex (male vs. female), dialect group (Hokkien vs. Cantonese), education level (no formal education, primary school, secondary or higher education), year of enrollment into the study (1993–1995, 1996–1998), BMI (<20, 20–23.9, 24–27.9, ≥28 kg/m^2^), tobacco smoking status (ever, never), alcohol intake status (heavy drinker, none or light drinker), history of diabetes (no, yes), family history of CRC (no, yes), and total daily energy intake (Kcal/day). In addition, we further adjusted for fiber in the model to evaluate the association between NSP and colorectal cancer risk and for NSP to evaluate the fiber–colorectal cancer association. Results are presented for colon and rectal cancer combined and separately. The same Cox regression model was used for subgroup analysis on subjects stratified by sex, BMI (<23 kg/m^2^ vs. ≥23 kg/m^2^), cigarette smoking (never vs. ever), and diabetes (no vs. yes). We also conducted sensitivity analysis by excluding colorectal cancer cases and person-years observed within the first two years of follow-up postenrollment.

SAS statistical package, version 9.4 (SAS Institute) was used for all statistical analysis. All *P* values cited are two sided. The *P* values less than 0.05 were considered statistically significant.

### Data Availability Statement

The limited dataset that supports the findings of this study is available on request from the corresponding author. The limited dataset is not publicly available due to privacy or ethical restrictions.

### Ethics Statement

All participants of the Singapore Chinese Health Study provided written consent forms. The Singapore Chinese Health Study has been approved by the Institutional Review Boards of the National University of Singapore and the University of Pittsburgh.

## Results

Overall, 2,140 colorectal cancer cases (1,355 colon cancer and 785 rectal cancer) were identified among the 61,321 participants who were free of cancer at baseline after a mean (SD) follow-up of 17.5 (5.4) years. The mean (SD) age of participants at the enrollment was 59.5 (8.0) years for colorectal cancer cases and 56.3 (8.0) years for those free of colorectal cancer.

Participants who developed colorectal cancer were older and more likely to be male, ever smokers, heavy drinkers, or to have a family history of CRC (All *P's* < 0.05; Table 1). Also, participants who developed colorectal cancer, on average, had a lower intake of dietary fiber and NSP compared with those who remained free of colorectal cancer (All *P's* ≤ 0.01).

**TABLE 1:** Distributions of baseline characteristics among study participants, The Singapore Chinese Healthy Study, 1993–2015

Characteristics	CRC Cases (*n* = 2,140)	Noncases (*n* = 59,181)	*P*
Age, mean (SD)^a^	59.5 (8.0)	56.3 (8.0)	<0.001
Sex, *n* (%)			
Male	1,115 (52.1)	26,178 (44.2)	<0.001
Female	1,025 (47.9)	33,003 (55.8)	
Dialect, *n* (%)			
Cantonese	960 (44.9)	27,365 (46.2)	0.21
Hokkien	1,180 (55.1)	31,816 (53.8)	
Highest level of education, *n* (%)			
No formal education	632 (29.5)	16,029 (27.1)	<0.001
Primary school	1,015 (47.4)	26,209 (44.3)	
Secondary school or higher	493 (23.1)	16,943 (28.6)	
Body mass index (kg/m^2^), mean (SD)	23.2 (3.3)	23.1 (3.3)	0.37
Smoking status, *n* (%)			
Never smoker	1,349 (63.0)	41,234 (69.7)	<0.001
Ever smoker	791 (37.0)	17,947 (30.3)	
Smoke pack per year, mean (SD)	11.0 (19.7)	8.9 (18.3)	<0.001
Alcohol consumption, *n* (%)			
Heavy drinker	56 (2.6)	951 (1.6)	<0.001
Non-heavy drinker	2,084 (97.4)	58,230 (98.4)	
Weekly physical activity^a^, *n* (%)			
No	1,438 (67.2)	39,645 (67.0)	0.84
Yes	702 (32.8)	19,536 (33.0)	
Had physician-diagnosed history of diabetes, *n* (%)			
No	1,942 (90.7)	53,910 (91.1)	0.58
Yes	198 (9.3)	5,271 (8.9)	
Had a family history of colorectal cancer, *n* (%)			
No	2,073 (96.9)	57,899 (97.8)	0.003
Yes	67 (3.1)	1,282 (2.2)	
Total energy intake (kcal/day), mean (SD)	1,559.1 (578.5)	1,556.5 (565.8)	0.83
Red meat/processed meat consumption, mean (SD)	2.5 (3.9)	2.7 (4.6)	0.05
Dietary fiber intake (g/day) mean (SD)	12.4 (5.8)	12.7 (5.8)	0.01
Dietary NSP intake (g/day) mean (SD)	8.0 (4.3)	8.4 (4.4)	<0.001
Insoluble NSP intake	4.4 (1.7)	4.6 (1.8)	<0.001
Soluble NSP intake	3.6 (1.7)	3.8 (1.7)	<0.001

^a^Including strenuous physical activity and/or vigorous work.

Higher intake of dietary fiber or NSP was observed more frequently in females, Cantonese origin, those who obtained secondary or higher education, had more physical activity, history of diabetes, higher energy consumption, but less likely to smoke cigarettes or drink alcohol heavily (Supplementary Table S2). Dietary fiber intake was highly correlated with dietary NSP with a correlation coefficient of 0.91 (*P* < 0.001).

The inverse associations between dietary fiber intake and risk of colorectal cancer or colon cancer were borderline significant after adjustment for other risk factors or potential confounders (HR_Q4 vs. Q1_ = 0.90, 95% CI: 0.79, 1.03, *P*_trend_ = 0.05)**.** These inverse fiber–cancer risk associations diminished after further adjustment for dietary NSP (HR_Q4 vs. Q1_ = 1.07, 95% CI: 0.85–1.34, *P*_trend_ = 0.77; Table 2). On the other hand, higher intake of dietary NSP was associated with significantly lower risk of colorectal cancer or colon cancer, ever after adjustment for dietary fiber and all other risk factors. Compared with the lowest quartile, the multivariate (including fiber)-adjusted HRs of colorectal cancer and colon cancer for the highest quartile of NSP were 0.80 (95% CI, 0.64–1.00) and 0.73 (95% CI, 0.55–0.97), respectively (both *P*_trend_ < 0.05; Table 2). The inverse association for NSP with risk of colorectal or colon cancer was similar as to those with soluble and insoluble NSP (Supplementary Table S3). Null association for either dietary fiber or NSP with risk of rectal cancer was also found.

**TABLE 2:** Association between dietary fibers and nonstarch polysaccharide intake and the risk of colorectal cancer in the Singapore Chinese Health Study, 1993–2015

	Q1	Q2	Q3	Q4	*P*_trend_
**Dietary fiber in quartile**					
Colorectal cancer					
Person-year	262,569	267,974	270,683	273,133	–
No. of cases	577	579	517	467	–
Multi-adjusted HR (95% CI)^a^	1.00	1.06 (0.94–1.19)	0.98 (0.86–1.10)	0.90 (0.79–1.03)	0.05
NSP-adjusted HR (95% CI)^b^	1.00	1.12 (0.98–1.28)	1.09 (0.92–1.30)	1.07 (0.85–1.34)	0.77
Colon cancer					
No. of cases	354	373	338	290	
Multi-adjusted HR (95% CI)^a^	1.00	1.06 (0.91–1.23)	0.98 (0.84–1.15)	0.86 (0.73–1.01)	0.03
NSP-adjusted HR (95% CI)^b^	1.00	1.15 (0.97–1.36)	1.16 (0.93–1.43)	1.10 (0.83–1.46)	0.70
Rectal cancer					
No. of cases	223	206	179	177	–
Multi-adjusted HR (95% CI)^a^	1.00	1.06 (0.87–1.29)	0.97 (0.79–1.19)	0.99 (0.80–1.21)	0.71
NSP-adjusted HR (95% CI)^b^	1.00	1.07 (0.86–1.34)	0.99 (0.74–1.32)	1.01 (0.70–1.47)	0.98
**Dietary Nonstarch polysaccharide in quartile**					
Colorectal cancer					
No. of cases	604	564	531	441	–
Multi-adjusted HR (95% CI)^a^	1.00	0.99 (0.88–1.11)	0.98 (0.87–1.11)	0.84 (0.73–0.95)	0.006
Fiber-adjusted HR (95% CI)^c^	1.00	0.97 (0.85–1.11)	0.95 (0.80–1.14)	0.80 (0.64–1.00)	0.03
Colon cancer					
No. of cases	378	369	331	277	
Multi-adjusted HR (95% CI)^a^	1.00	0.98 (0.85–1.14)	0.93 (0.79–1.08)	0.79 (0.67–0.93)	0.003
Fiber-adjusted HR (95% CI)^c^	1.00	0.96 (0.81–1.13)	0.88 (0.71–1.09)	0.73 (0.55–0.97)	0.02
Rectal cancer					
No. of cases	226	195	200	164	–
Multi-adjusted HR (95% CI)^a^	1.00	1.00 (0.82–1.22)	1.10 (0.90–1.34)	0.92 (0.74–1.13)	0.53
Fiber-adjusted HR (95% CI)^c^	1.00	1.00 (0.80–1.26)	1.11 (0.83–1.47)	0.93 (0.64–1.35)	0.59

^a^Model adjusted for age, sex, dialect, year of interview, education level, BMI levels (<18.5, 18.5–<23.0, 23.0–<27.0, ≥27.0), smoking status, alcohol consumption, history of diabetes, physical activity, family history of colorectal cancer, red meat/processed meat consumption, smoke pack per year, and total energy intake.

^b^Model further adjusted for NSP.

^c^Model further adjusted for dietary fiber intake.

In the subgroup analysis by sex, BMI, smoking status, and history of diabetes, a statistically significant inverse association for dietary NSP with colorectal cancer and colon cancer risk was observed only in men, individuals with BMI > 23 kg/m^2^, or those without a history of diabetes (all *P*_trend_ < 0.05; Table 3). We conducted similar subgroup analysis which did not reveal consistent association for dietary fiber with the risk of colon or rectal cancer or combined (Supplementary Table S4).

**TABLE 3:** Risk of colorectal cancer in relation to dietary nonstarch polysaccharide intake levels, the Singapore Chinese Health Study, 1993–2015^a^

		Q1	Q2	Q3	Q4	*P*_trend_	*P*_interaction_
**CRC risk in subgroup**							
	Cases, *n*	402	284	225	204		
Men	HR (95% CI)	1.00	1.07 (0.92–1.25)	0.94 (0.80–1.12)	**0.84 (0.70–1.00)**	**0.03**	0.74
	Cases, *n*	202	280	306	237		
Women	HR (95% CI)	1.00	0.90 (0.75–1.08)	1.00 (0.83–1.20)	**0.82 (0.68–1.00)**	0.09	
	Cases, *n*	304	262	264	198		
BMI < 23	HR (95% CI)	1.00	1.00 (0.84–1.18)	1.10 (0.93–1.31)	0.84 (0.69–1.01)	0.12	0.71
	Cases, *n*	300	302	267	243		
BMI ≥ 23	HR (95% CI)	1.00	0.98 (0.83–1.15)	0.88 (0.75–1.05)	**0.83 (0.69–0.99)**	**0.02**	
	Cases, *n*	283	365	374	327		
Never smokers	HR (95% CI)	1.00	1.00 (0.85–1.17)	1.00 (0.86–1.17)	0.86 (0.73–1.01)	0.05	0.76
	Cases, *n*	321	199	157	114		
Ever smokers	HR (95% CI)	1.00	0.99 (0.82–1.19)	0.97 (0.80–1.18)	0.81 (0.65–1.01)	0.08	
	Cases, *n*	563	499	482	398		
No history of DM	HR (95% CI)	1.00	0.96 (0.85–1.09)	0.98 (0.86–1.11)	**0.85 (0.74–0.97)**	**0.02**	0.41
	Cases, *n*	41	65	49	43		
DM history	HR (95% CI)	1.00	1.33 (0.89–1.99)	1.05 (0.69–1.62)	0.77 (0.50–1.20)	0.07	
**Colon cancer risk in subgroups**							
	Cases, *n*	242	169	124	121		
Men	HR (95% CI)	1.00	1.02 (0.84–1.25)	0.82 (0.65–1.02)	**0.76 (0.61–0.96)**	**0.008**	0.51
	Cases, *n*	136	200	207	156		
Women	HR (95% CI)	1.00	0.95 (0.76–1.18)	1.00 (0.80–1.25)	0.82 (0.64–1.04)	0.10	
	Cases, *n*	180	163	162	120		
BMI < 23	HR (95% CI)	1.00	0.98 (0.79–1.22)	1.06 (0.85–1.32)	**0.78 (0.61–0.99)**	0.07	0.60
	Cases, *n*	198	206	169	157		
BMI ≥ 23	HR (95% CI)	1.00	0.98 (0.80–1.19)	0.82 (0.67–1.02)	**0.80 (0.64–0.99)**	**0.02**	
	Cases, *n*	190	259	250	210		
Never smokers	HR (95% CI)	1.00	1.05 (0.87–1.27)	1.00 (0.83–1.21)	0.83 (0.68–1.02)	**0.03**	0.68
	Cases, *n*	188	110	81	67		
Ever smokers	HR (95% CI)	1.00	0.90 (0.70–1.14)	0.81 (0.62–1.06)	0.77 (0.58–1.03)	0.05	
	Cases, *n*	349	325	302	249		
No history of DM	HR (95% CI)	1.00	0.95 (0.82–1.12)	0.93 (0.80–1.10)	**0.81 (0.68–0.96)**	**0.02**	0.31
	Cases, *n*	29	44	29	28		
DM history	HR (95% CI)	1.00	1.24 (0.77–2.02)	0.84 (0.49–1.43)	0.65 (0.38–1.11)	**0.03**	
**Rectal cancer risk in subgroups**							
	Cases, *n*	160	115	101	83		
Men	HR (95% CI)	1.00	1.15 (0.90–1.47)	1.16 (0.89–1.50)	0.96 (0.73–1.27)	0.92	0.90
	Cases, *n*	66	80	99	81		
Women	HR (95% CI)	1.00	0.81 (0.58–1.12)	0.99 (0.72–1.35)	0.83 (0.59–1.16)	0.52	
	Cases, *n*	124	99	102	78		
BMI < 23	HR (95% CI)	1.00	1.03 (0.78–1.35)	1.19 (0.90–1.56)	0.94 (0.70–1.27)	0.88	0.91
	Cases, *n*	102	96	98	86		
BMI ≥ 23	HR (95% CI)	1.00	0.97 (0.73–1.29)	1.01 (0.76–1.35)	0.89 (0.66–1.20)	0.48	
	Cases, *n*	93	106	124	117		
Never smokers	HR (95% CI)	1.00	0.89 (0.67–1.18)	1.01 (0.76–1.33)	0.92 (0.69–1.21)	0.73	0.84
	Cases, *n*	133	89	76	47		
Ever smokers	HR (95% CI)	1.00	1.13 (0.86–1.49)	1.22 (0.91–1.63)	0.86 (0.61–1.21)	0.69	
	Cases, *n*	214	174	180	149		
No history of DM	HR (95% CI)	1.00	0.97 (0.79–1.19)	1.07 (0.87–1.31)	0.91 (0.73–1.14)	0.54	0.99
	Cases, *n*	12	21	20	15		
DM history	HR (95% CI)	1.00	1.53 (0.74–3.19)	1.61 (0.77–3.37)	1.08 (0.49–2.36)	0.92	

Abbreviations: CRC, Colorectal cancer; DM, diabetes mellitus.

^a^Model adjusted for age, sex, dialect, year of interview, education level, physical activity, family history of colorectal cancer, and total energy intake, as well as history of diabetes, BMI level, smoking status, alcohol consumption, red meat/processed meat consumption, smoke pack per year, if applicable.

In the sensitivity analysis, we excluded colorectal cancer cases and person-years observed within the first two years of follow-up and found similar estimates: high levels of dietary NSP intake were inversely associated with risk of colorectal cancer (HR_Q4 vs. Q1_ = 0.84, 95% CI: 0.74, 0.96, *P*_trend_ = 0.009) and colon cancer (HR_Q4 vs. Q1_ = 0.81; 95% CI: 0.68–0.96; *P*_trend_ = 0.006), but was not associated with rectal cancer (HR_Q4 vs. Q1_ = 0.91; 95% CI: 0.73, 1.13; *P*_trend_ = 0.47). Similarly, dietary fiber was associated with lower risk of colon cancer (*P*_trend_ = 0.04), but not with rectal cancer (*P*_trend_ = 0.60; Supplementary Table S5).

## Discussion

In the current analysis of an ongoing prospective cohort study including 61,321 Chinese men and women in Singapore, we showed that a higher intake of NSP was associated with a lower risk of colorectal cancer and colon cancer even after adjustment for dietary fiber. The inverse association was robust and consistent across different subgroups. On the other hand, the inverse association between dietary fiber and colorectal cancer isk maybe explained by the dietary NSP in this study.

To our knowledge, this is the first prospective cohort study that evaluated the association of both dietary fiber and NSP with colorectal cancer risk in Chinese adults. Previous studies focus on dietary fiber in Western populations. The NHS (17), a prospective study of 88,757 US women participants with 16 years of follow-up, found no strong evidence to support that dietary fiber is protective against colorectal cancer. In the HPFS (18), 47,949 U.S. men were followed 6 years, no association between fiber or vegetable intake and the risk of colorectal cancer were identified. A similar null association between dietary fiber intake and colorectal cancer was also reported from the Alpha-Tocopherol, Beta-Carotene Cancer Prevention Study in Finland among 27,111 male smokers with an average follow-up of 8 years (20). However, a high-level intake of all vegetable and dietary fiber was associated with a lower risk of colorectal cancer in the Iowa Women's Health Study, a prospective cohort study of (41,837 U.S. women with 5 years of follow-up, in the EPIC study; 519,978 Europeans with 4.5 years of follow-up on average; ref. 26) and in the MEC study (85,903 U.S. men and 105,108 U.S. women with an average of 7.3 years of follow-up; ref. 19). A meta-analysis summarized 13 case–control studies with 5,287 cases of colorectal cancer and 10,470 control concluded fiber-rich foods may decrease the risk of colorectal cancer (39). These previous studies did not adjust for dietary NSP intake while examining the effects of dietary fiber and the inconsistency of these studies is likely due to different adjustments of covariates, follow-up time, or different types of fiber.

As for dietary NSP, available epidemiologic studies are sparse and reported to be inversely related to the risk of colon cancer such as about to 3–4 fold differences in incidence of large bowel cancer among four Scandinavian populations (120 men in total; ref. 40). A meta-analysis reported that NSP has an inverse association with the risk of colorectal cancer by summarizing data from 12 different countries (24), although some of the studies included in this meta-analysis refer to dietary fiber as NSP. The lack of study focused on dietary NSP and the risk of colorectal cancer is likely due to the difficulties of distinguishing dietary fiber from dietary NSP.

Dietary NSP is found naturally in many foods (41) and has corresponding properties with dietary fiber (42) such as laxative effects (24). The insoluble NSP (cellulose and hemicellulose) provides the laxative effects and the soluble NSP (mixed-link β-glucans) may lower the cholesterol level and maintain healthy glucose and insulin levels (41). We found the inverse association for NSP intake was primarily for colon cancer, but not for rectal cancer. The biology behind the protective effect of NSP is not fully understood, but undigested NSP, when partially fermented by colon bacteria produces short-chain fatty acids (propionate, acetate, and butyrate), and increases the stool weight and decreases the transit time of feces in the colon (24), all thought to be protective against colorectal cancer. On the other hand, dietary fiber was not protective against the development of colorectal cancer, which is consistent with the null results by the clinical trial studies (43–45, 28).

As a subtype of dietary fiber, NSP is the fraction of carbohydrates excluding starch and free sugar molecules (46, 47). The other types of dietary fiber, including resistant starch and oligosaccharides, may provide other health benefits such as reducing the risk of type 2 diabetes (48), but may not offer a protective effect for colorectal cancer. This differential protective effect may be due to the sources of the food since NSPs are mainly present in primary or secondary plant cell walls (49). Surrounded by growing plant cells, primary cell walls consist of polysaccharide and structural proteins while secondary cell walls, in which mature plant cells are surrounded, contain lignin and cellulose in addition to polysaccharide and structural proteins (50). However, foods contain different types of fiber or nutrients, which is difficult to separate out (51). Future studies are warranted to determine the biological mechanisms for the differential effects of dietary NSP intake on colorectal cancer prevention.

The present study had several strengths. This was the first prospective study examining the association between dietary fiber or NSP and the risk of colorectal cancer in an Asian population. As a prospective study, information on dietary, supplement, and related lifestyle factors were collected prior to diagnosis of colorectal cancer, minimizing the potential impact of colorectal cancer diagnosis on dietary habits or lifestyle factors. We were able to comprehensively evaluate both dietary fiber and dietary NSP while monitoring for potential contributing factors. Sufficient statistical power is provided by the large sample size with long-term follow-up.

Our study also had several limitations. The FFQ at baseline only captured a one-time assessment of dietary pattern among participants while the dietary pattern can change over time. Changes in the dietary pattern over time after the baseline dietary assessment might result in non-differential misclassification of both colorectal cancer cases and noncases, which could lead to the underestimation of the dietary exposure–colorectal cancer risk association toward the null (52). In addition, endoscopic screening information was not collected in the SCHS, although there was no national colorectal cancer screening program in place when the cohort was assembled. The FFQ, including fiber component was validated by a series of 24-hour food recalls. Although we do not have specific biomarkers for NSP, it is however noted that NSP was highly correlated with fiber in our study population (*r* = 0.92; ref. 30). With multiple lifestyle factors examined, residual confounding may persist. In addition, there were small percentage of participants used calcium supplements (3.4% at baseline) or calcium use (5.06% at follow-up 1), therefore, assessing the potential confounding effect by calcium supplement use or aspirin use is not applicable.

In summary, this is the first prospective study that examined simultaneous dietary fiber and NSP with the risk of colorectal cancer in an Asian population and revealed that dietary NSP rather than fiber was statistically significantly associated with a lower risk of colorectal cancer. The potential protective effect of NSP, independent of dietary fiber, on colorectal cancer development warrants further study.

## Supplementary Material

Supplementary Tables 1-5 Supplementary Table 1. Distributions of baseline characteristics among study participants by levels of dietary fibers and non-starch polysaccharide intake, the Singapore Chinese Health Study, 1993-2015. Supplementary Table 2. Association between soluble, insoluble non-starch polysaccharide intake with the risk of colorectal cancer in the Singapore Chinese Health Study, 1993-2015. Supplementary Table 3. Risk of colorectal cancer in relation to dietary fiber intake levels, the Singapore Chinese Health Study, 1993-2015. Supplementary Table 4. Association between dietary fibers intake and dietary non-starch polysaccharide with the risk of colorectal cancer in the Singapore Chinese Health Study, 1993-2015, excluding cases and person-years within the first 2 years of observation. Supplementary Table 5. Food Composition Table of dietary fiber, soluble non-starch polysaccharide, and insoluble non-starch polysaccharide: The Singapore Chinese Health Study.
