# Supplementary material for: Dietary Nonstarch Polysaccharide Intake and Risk of Colorectal Cancer: Findings from the Singapore Chinese Health Study
Source: Cancer Res Commun. 2022 Oct 31;2(10):1304–11. doi: 10.1158/2767-9764.CRC-22-0153 (PMC9683694; doi:10.1158/2767-9764.CRC-22-0153)
Supplement: Supplementary Tables 1-5 — Supplementary Table 1. Distributions of baseline characteristics among study participants by levels of dietary fibers and non-starch polysaccharide intake, the Singapore Chinese Health Study, 1993-2015. Supplementary Table 2. Association between soluble, insoluble non-starch polysaccharide intake with the risk of colorectal cancer in the Singapore Chinese Health Study, 1993-2015. Supplementary Table 3. Risk of colorectal cancer in relation to dietary fiber intake levels, the Singapore Chinese Health Study, 1993-2015. Supplementary Table 4. Association between dietary fibers intake and dietary non-starch polysaccharide with the risk of colorectal cancer in the Singapore Chinese Health Study, 1993-2015, excluding cases and person-years within the first 2 years of observation. Supplementary Table 5. Food Composition Table of dietary fiber, soluble non-starch polysaccharide, and insoluble non-starch polysaccharide: The Singapore Chinese Health Study. [file crc-22-0153-s01.docx]

**Dietary Non-starch Polysaccharide Intake and Risk of Colorectal Cancer:**

**Findings from the Singapore Chinese Health Study**

**Supplementary Tables**

Yi-Chuan Yu^1^, Pedram Paragomi^1^, Aizhen Jin^2^, Renwei Wang^1^,

Robert E. Schoen^3,4^, Woon-Puay Koh^2,5^, Jian-Min Yuan^1,4^, Hung N. Luu^1,4^

**Supplementary Table 1. Food groups for classifying dietary fiber, insoluble non-starch polysaccharide, and soluble non-starch polysaccharide: The Singapore Chinese Health Study**

|  | Dietary Fiber (%) | Insoluble NSP (%) | Soluble NSP (%) |
| --- | --- | --- | --- |
| All red meat | 0.91 | 0.88 | 0.93 |
| Poultry | 0.18 | 0.22 | 0.20 |
| All fish and shellfish | 1.22 | 0.99 | 1.08 |
| Eggs | 0.20 | 0.24 | 0.22 |
| Tofu products and soybean drink | 3.35 | 6.13 | 4.76 |
| Non-soy legumes | 0.82 | 0.87 | 0.78 |
| Green vegetables | 12.68 | 17.43 | 14.66 |
| Cruciferous vegetables | 8.75 | 11.80 | 10.13 |
| Yellow-orange vegetables | 1.32 | 1.61 | 1.70 |
| White potatoes | 0.82 | 0.62 | 1.05 |
| Tomato products | 1.29 | 1.34 | 1.14 |
| Other vegetables | 2.34 | 2.10 | 1.78 |
| Preserved vegetables | 2.08 | 0.08 | 0.08 |
| Citrus fruits and juices | 8.65 | 7.40 | 18.20 |
| Other selected fruits and/or juice | 16.67 | 19.86 | 21.91 |
| Other fruits, fruit juices, or dried fruits | 2.26 | 0.80 | 0.52 |
| Noodles and pasta | 7.69 | 7.13 | 7.03 |
| Rice | 8.00 | 7.76 | 0.32 |
| Bread and pancakes | 10.82 | 11.06 | 9.62 |
| Breakfast cereals | 2.35 | 0.95 | 1.94 |
| Biscuits and crackers | 1.73 | 1.20 | 2.10 |
| Dairy products | 0.11 | 0.05 | 0.06 |
| Desserts | 0.59 | 0.79 | 0.75 |
| Nuts and seeds | 1.26 | 1.63 | 0.85 |
| Bread spread | 0.40 | 0.66 | 0.37 |
| Cooking fats/oil | 0.48 | 0.48 | 0.44 |
| Sugar and candy | 0.39 | 0.40 | 0.26 |
| Non-dairy beverages | 0.51 | 0.00 | 0.00 |
| Sauces and condiments | 1.73 | 1.41 | 2.32 |
| Flour, baking ingredients | 0.75 | 0.59 | 0.48 |
| Soup stock, canned soup | 1.64 | 1.46 | 1.55 |

**Supplementary Table 2. Distributions of baseline characteristics among study participants**

**by levels of dietary fibers and non-starch polysaccharide intake, the Singapore Chinese Health Study, 1993-2015**

|  | **Dietary Fiber Intake** | | | **Dietary Non-starch Polysaccharide Intake** | | |
| --- | --- | --- | --- | --- | --- | --- |
|  | **Quartile 1** | **Quartile 4** | ***P*** | **Quartile 1** | **Quartile4** | ***P*** |
| Mean age (±SD), years | 56.5 (7.9) | 55.9 (7.9) | <0.001 | 57.0 (8.0) | 55.4 (7.7) | <0.001 |
| Mean BMI (±SD), Kg/m^2^ | 23.0 (3.3) | 23.2 (3.3) | 0.49 | 23.0 (3.3) | 23.2 (3.3) | 0.56 |
| Mean total energy intake (±SD), Kcal | 1,700.9 (624.7) | 1,648.3 (555.2) | <0.001 | 1,718.1 (615.7) | 1,652.2 (568.7) | <0.001 |
| Mean red meat/processed meat consumption, mean (±SD) | 2.7 (5.3) | 2.4 (4.7) | <0.001 | 2.5 (5.0) | 2.5 (4.9) | <0.001 |
| Sex (N, %) |  |  |  |  |  |  |
| Male | 9,691 (62.7) | 5,757 (35.9) | <0.001 | 9,630 (62.5) | 5,661 (37.1) | <0.001 |
| Female | 5,757 (37.3) | 9,747 (64.1) |  | 5,790 (37.5) | 9,600 (62.9) |  |
| Dialect (N, %) |  |  |  |  |  |  |
| Cantonese | 6,824 (44.2) | 7,466 (49.1) | <0.001 | 6,591 (42.7) | 7,869 (51.6) | <0.001 |
| Hokkien | 8,624 (55.8) | 7,738 (50.9) |  | 8,829 (57.3) | 7,392 (48.4) |  |
| Highest level of education (N, %) |  |  |  |  |  |  |
| No formal education | 4,357 (28.2) | 3,256 (21.5) | <0.001 | 4,588 (29.7) | 2,933 (19.2) | <0.001 |
| Primary school | 7,536 (48.8) | 6,255 (41.1) |  | 7,533 (48.9) | 6,275 (41.1) |  |
| Secondary school or higher | 3,555 (23.0) | 5,693 (37.4) |  | 3,299 (21.4) | 6,053 (39.7) |  |
| Mean smoke pack per year, mean (±SD) | 15.7 (22.8) | 5.1 (14.5) | <0.001 | 16.0 (23.0) | 5.2 (14.4) | <0.001 |
| Smoking status (N, %) |  |  |  |  |  |  |
| Never smoker | 7,996 (51.8) | 12,236 (80.5) | <0.001 | 7,950 (51.6) | 12,177 (79.8) | <0.001 |
| Ever smoker | 7,452 (48.2) | 2,968 (19.5) |  | 7,470 (48.4) | 3,084 (20.2) |  |
| Alcohol consumption (N, %) |  |  |  |  |  |  |
| Heavy drinker | 686 (4.4) | 73 (0.5) | <0.001 | 770 (5.0) | 70 (0.5) | <0.001 |
| Non-heavy drinker | 14,762 (95.6) | 15,131 (99.5) |  | 14,650 (95.0) | 15,191 (99.5) |  |
| Weekly physical activity (N, %) |  |  |  |  |  |  |
| No | 10,681 (69.1) | 9,047 (59.5) | <0.001 | 10,647 (69.0) | 9,067 (59.4) | <0.001 |
| Yes | 4,767 (30.9) | 6,157 (40.5) |  | 4,773 (31.0) | 6,194 (40.6) |  |
| History of diabetes (N, %) |  |  |  |  |  |  |
| No | 14,324 (92.7) | 13,682 (90.0) | <0.001 | 14,266 (92.5) | 13,750 (90.1) | <0.001 |
| Yes | 1,124 (7.3) | 1,522 (10.0) |  | 1,154 (7.5) | 1,511 (9.9) |  |
| Family history of CRC (N, %) |  |  |  |  |  |  |
| No | 15,144 (98.0) | 14,803 (97.4) | <0.001 | 15,139 (98.2) | 14,853 (97.3) | <0.001 |
| Yes | 304 (2.0) | 401 (2.6) |  | 281 (1.8) | 408 (2.7) |  |

Abbreviations: CRC: colorectal cancer; SD: standard deviation

Weekly physical activity: including strenuous physical activity and/or vigorous work

**Supplementary Table 3. Association between soluble, insoluble non-starch polysaccharide intake with the risk of colorectal cancer in the Singapore Chinese Health Study, 1993-2015**

| **Insoluble Non-starch Polysaccharide in quartile** | | | | | | |
| --- | --- | --- | --- | --- | --- | --- |
|  | **Q1** | **Q2** | | **Q3** | **Q4** | ***P_trend_*** |
| **Colorectal Cancer** | | | | | | |
| Person-year | 258,635 | 266,338 | | 273,213 | 276,174 |  |
| No. of cases | 612 | 549 | | 529 | 450 |  |
| HR (95% CI)* | 1.00 | 0.95 (0.84, 1.07) | | 0.97 (0.86, 1.10) | **0.84 (0.74, 0.96)** | **0.01** |
| **Colon Cancer** | | | | | | |
| No. of cases | 380 | 368 | | 321 | 286 |  |
| HR (95% CI)* | 1.00 | 0.98 (0.84, 1.13) | | 0.90 (0.77, 1.05) | **0.82 (0.70, 0.96)** | **0.008** |
| **Rectal Cancer** |  |  | |  |  |  |
| No. of cases | 232 | 181 | | 208 | 164 |  |
| HR (95% CI)* | 1.00 | 0.90 (0.73, 1.10) | | 1.11 (0.92, 1.36) | 0.89 (0.72, 1.10) | 0.56 |
| **Soluble Non-starch Polysaccharide in quartile** | | | | | | |
|  | **Q1** | **Q2** | **Q3** | | **Q4** | ***P_trend_*** |
| **Colorectal Cancer** | | | | | | |
| No. of cases | 598 | 566 | 523 | | 453 |  |
| HR (95% CI)* | 1.00 | 0.99 (0.88, 1.12) | 0.97 (0.86, 1.10) | | **0.87 (0.76, 0.99)** | **0.03** |
| **Colon Cancer** | | | | | | |
| No. of cases | 375 | 375 | 322 | | 283 |  |
| HR (95% CI)* | 1.00 | 0.99 (0.86, 1.15) | 0.90 (0.77, 1.05) | | **0.82 (0.70, 0.96)** | **0.008** |
| **Rectal Cancer** |  |  |  | |  |  |
| No. of cases | 223 | 191 | 201 | | 170 |  |
| HR (95% CI)* | 1.00 | 0.98 (0.80, 1.20) | 1.11 (0.91, 1.35) | | 0.96 (0.78, 1.19) | 0.92 |

*Model adjusted for age, sex, dialect, year of interview, education level, BMI levels (<18.5, 18.5-<23.0, 23.0-<27.0, ≥27.0), smoking status, alcohol consumption, history of diabetes, physical activity, family history of colorectal cancer, red meat/processed meat consumption, smoke pack per year, and total energy intake

**Supplementary Table 4. Risk of colorectal cancer in relation to dietary fiber intake levels,**

**the Singapore Chinese Health Study, 1993-2015**^*^

|  |  | **Q1** | **Q2** | **Q3** | **Q4** | ***P_Trend_*** | ***P_Interaction_*** |
| --- | --- | --- | --- | --- | --- | --- | --- |
| **CRC risk in subgroup** | | | | | | | |
|  | No. Cases | 405 | 279 | 222 | 209 |  |  |
| Men | HR (95% CI) | 1.00 | 1.03 (0.88, 1.20) | 0.89 (0.75, 1.05) | 0.86 (0.72, 1.02) | 0.05 | 0.37 |
|  | No. Cases | 172 | 300 | 295 | 258 |  |  |
| Women | HR (95% CI) | 1.00 | 1.14 (0.94, 1.38) | 1.10 (0.91, 1.33) | 0.98 (0.80, 1.20) | 0.44 |  |
|  | No. Cases | 298 | 251 | 262 | 217 |  |  |
| BMI<23 | HR (95% CI) | 1.00 | 0.97 (0.82, 1.15) | 1.07 (0.90, 1.27) | 0.91 (0.75, 1.09) | 0.42 | 0.43 |
|  | No. Cases | 279 | 328 | 255 | 250 |  |  |
| BMI≥23 | HR (95% CI) | 1.00 | 1.14 (0.97, 1.34) | 0.90 (0.75, 1.07) | 0.90 (0.75, 1.07) | 0.05 |  |
|  | No. Cases | 257 | 379 | 368 | 345 |  |  |
| Never smokers | HR (95% CI) | 1.00 | 1.15 (0.98, 1.35) | 1.06 (0.90, 1.24) | 0.95 (0.80, 1.12) | 0.17 | 0.61 |
|  | No. Cases | 320 | 200 | 149 | 122 |  |  |
| Ever smokers | HR (95% CI) | 1.00 | 0.97 (0.81, 1.17) | 0.89 (0.73, 1.09) | 0.88 (0.71, 1.10) | 0.18 |  |
|  | No. Cases | 533 | 523 | 465 | 421 |  |  |
| No DM history | HR (95% CI) | 1.00 | 1.06 (0.94, 1.20) | 0.98 (0.86, 1.11) | 0.92 (0.80, 1.05) | 0.12 | 0.42 |
|  | No. Cases | 44 | 56 | 52 | 46 |  |  |
| DM history | HR (95% CI) | 1.00 | 1.09 (0.72, 1.64) | 0.96 (0.64, 1.45) | 0.76 (0.50, 1.17) | 0.12 |  |
| **Colon cancer risk in subgroups** | | | | | | | |
|  | No. Cases | 238 | 160 | 139 | 119 |  |  |
| Men | HR (95% CI) | 1.00 | 0.97 (0.79, 1.19) | 0.90 (0.73, 1.12) | **0.77 (0.61, 0.96)** | **0.02** | 0.44 |
|  | No. Cases | 116 | 213 | 199 | 171 |  |  |
| Women | HR (95% CI) | 1.00 | 1.19 (0.95, 1.49) | 1.10 (0.87, 1.38) | 0.98 (0.77, 1.24) | 0.39 |  |
|  | No. Cases | 172 | 157 | 164 | 132 |  |  |
| BMI<23 | HR (95% CI) | 1.00 | 0.99 (0.79, 1.23) | 1.07 (0.85, 1.33) | 0.86 (0.67, 1.09) | 0.27 | 0.36 |
|  | No. Cases | 182 | 216 | 174 | 158 |  |  |
| BMI≥23 | HR (95% CI) | 1.00 | 1.12 (0.91, 1.37) | 0.91 (0.74, 1.13) | 0.86 (0.69, 1.07) | 0.05 |  |
|  | No. Cases | 175 | 268 | 243 | 223 |  |  |
| Never smokers | HR (95% CI) | 1.00 | 1.19 (0.98, 1.44) | 1.02 (0.84, 1.25) | 0.91 (0.74, 1.11) | 0.07 | 0.87 |
|  | No. Cases | 179 | 105 | 95 | 67 |  |  |
| Ever smokers | HR (95% CI) | 1.00 | 0.88 (0.69, 1.12) | 0.98 (0.76, 1.26) | 0.82 (0.62, 1.10) | 0.26 |  |
|  | No. Cases | 327 | 334 | 302 | 262 |  |  |
| No DM history | HR (95% CI) | 1.00 | 1.05 (0.90, 1.23) | 0.98 (0.83, 1.15) | 0.88 (0.74, 1.04) | 0.09 | 0.48 |
|  | No. Cases | 27 | 39 | 36 | 28 |  |  |
| DM history | HR (95% CI) | 1.00 | 1.21 (0.73, 2.01) | 1.05 (0.63, 1.75) | 0.70 (0.40, 1.20) | 0.09 |  |
| **Rectal cancer risk in subgroups** | | | | | | | |
|  | No. Cases | 167 | 119 | 83 | 90 |  |  |
| Men | HR (95% CI) | 1.00 | 1.11 (0.87, 1.41) | 0.87 (0.66, 1.14) | 1.01 (0.77, 1.32) | 0.76 | 0.57 |
|  | No. Cases | 56 | 87 | 96 | 87 |  |  |
| Women | HR (95% CI) | 1.00 | 1.03 (0.74, 1.45) | 1.11 (0.79, 1.55) | 0.99 (0.70, 1.40) | 0.92 |  |
|  | No. Cases | 126 | 94 | 98 | 85 |  |  |
| BMI<23 | HR (95% CI) | 1.00 | 0.95 (0.72, 1.25) | 1.07 (0.81, 1.41) | 0.99 (0.74, 1.33) | 0.90 | 0.92 |
|  | No. Cases | 97 | 112 | 81 | 92 |  |  |
| BMI≥23 | HR (95% CI) | 1.00 | 1.18 (0.89, 1.57) | 0.87 (0.64, 1.18) | 0.99 (0.73, 1.33) | 0.53 |  |
|  | No. Cases | 82 | 111 | 125 | 122 |  |  |
| Never smokers | HR (95% CI) | 1.00 | 1.07 (0.80, 1.43) | 1.13 (0.85, 1.50) | 1.04 (0.78, 1.39) | 0.86 | 0.25 |
|  | No. Cases | 141 | 95 | 54 | 55 |  |  |
| Ever smokers | HR (95% CI) | 1.00 | 1.10 (0.84, 1.44) | 0.77(0.56, 1.06) | 0.97 (0.70, 1.34) | 0.45 |  |
|  | No. Cases | 206 | 189 | 163 | 159 |  |  |
| No DM history | HR (95% CI) | 1.00 | 1.08 (0.88, 1.32) | 0.98 (0.79, 1.22) | 0.99 (0.80, 1.24) | 0.77 | 0.69 |
|  | No. Cases | 17 | 17 | 16 | 18 |  |  |
| DM history | HR (95% CI) | 1.00 | 0.87 (0.43, 1.74) | 0.81 (0.40, 1.64) | 0.90 (0.45, 1.79) | 0.79 |  |

^*^Model adjusted for age, sex, dialect, year of interview, education level, physical activity, family history of colorectal cancer, and total energy intake, as well as history of diabetes, BMI level, smoking status, alcohol consumption, red meat/processed meat consumption, smoke pack per year, if applicable.

Abbreviation: DM: diabetes mellitus

**Supplementary Table 5. Association between dietary fibers intake and dietary non-starch polysaccharide with the risk of colorectal cancer in the Singapore Chinese Health Study, 1993-2015**

**excluding cases and person-years within the first 2 years of observation**

|  | **Colorectal cancer** | | | **Colon Cancer** | | **Rectal Cancer** | |
| --- | --- | --- | --- | --- | --- | --- | --- |
|  | **Person-year** | **Cases** | **HR^a^ (95% CI)** | **Cases** | **HR^a^ (95% CI)** | **Cases** | **HR^a^ (95% CI)** |
| **Fiber intake in quartile** | | | | | | | |
| Q1 | 231,962 | 544 | 1.00 | 334 | 1.00 | 210 | 1.00 |
| Q2 | 237,486 | 544 | 1.06 (0.94, 1.20) | 353 | 1.07 (0.92, 1.25) | 191 | 1.04 (0.85, 1.27) |
| Q3 | 240,336 | 492 | 0.99 (0.87, 1.12) | 321 | 0.99 (0.85, 1.16) | 171 | 0.97 (0.79, 1.20) |
| Q4 | 242,956 | 437 | 0.89 (0.78, 1.02) | 274 | 0.86 (0.73, 1.02) | 163 | 0.96 (0.77, 1.19) |
| *P_trend_* |  |  | 0.05 |  | **0.04** |  | 0.60 |
| **Non-starch Polysaccharide**  **Intake in quartile** | | | | | | | |
| Q1 | 228,604 | 565 | 1.00 | 354 | 1.00 | 211 | 1.00 |
| Q2 | 236,216 | 537 | 1.01 (0.89, 1.14) | 353 | 1.01 (0.87, 1.17) | 184 | 1.00 (0.82, 1.23) |
| Q3 | 241,558 | 497 | 0.98 (0.87, 1.11) | 310 | 0.93 (0.79, 1.09) | 187 | 1.09 (0.88, 1.33) |
| Q4 | 246,362 | 418 | **0.84 (0.74, 0.96)** | 265 | **0.81 (0.68, 0.96)** | 153 | 0.91 (0.73, 1.13) |
| *P_trend_* |  |  | **0.009** |  | **0.006** |  | 0.47 |

^a^Model adjusted for age, sex, dialect, year of interview, education level, BMI levels (<18.5, 18.5-<23.0, 23.0-<27.0, ≥27.0), smoking status, alcohol consumption, history of diabetes, physical activity, red meat/processed meat consumption, smoke pack per year, and total energy intake for total subjects
